# Barriers and Drivers Regarding the Use of Mobile Health Apps Among Patients With Type 2 Diabetes Mellitus in the Netherlands: Explanatory Sequential Design Study

**DOI:** 10.2196/31451

**Published:** 2022-01-27

**Authors:** Marloes Bults, Catharina Margaretha van Leersum, Theodorus Johannes Josef Olthuis, Robin Enya Marije Bekhuis, Marjolein Elisabeth Maria den Ouden

**Affiliations:** 1 Technology, Health & Care Research Group Saxion University of Applied Sciences Enschede Netherlands; 2 Science, Technology, and Policy Studies Faculty of Behavioural, Management and Social Sciences University of Twente Enschede Netherlands; 3 Care & Technology Research Group Regional Community College of Twente Hengelo Netherlands; 4 Department of Smartup Innovation Ziekenhuisgroep Twente Almelo Netherlands

**Keywords:** type 2 diabetes, self-management, mobile health, mobile apps, prevention, mixed methods research, acceptance, mobile phone

## Abstract

**Background:**

Self-monitoring of blood glucose levels, food intake, and physical activity supports self-management of patients with type 2 diabetes mellitus (T2DM). There has been an increase in the development and availability of mobile health apps for T2DM.

**Objective:**

The aim of this study is to explore the actual use of mobile health apps for diabetes among patients with T2DM and the main barriers and drivers among app users and nonusers.

**Methods:**

An explanatory sequential design was applied, starting with a web-based questionnaire followed by semistructured in-depth interviews. Data were collected between July and December 2020. Questionnaire data from 103 respondents were analyzed using IBM SPSS Statistics (version 25.0). Descriptive statistics were performed for the actual use of apps and items of the Unified Theory of Acceptance and Use of Technology (UTAUT). The UTAUT includes 4 key constructs: performance expectancy (the belief that an app will help improve health performance), effort expectancy (level of ease associated with using an app), social influence (social support), and facilitating conditions (infrastructural support). Differences between users and nonusers were analyzed using chi-square tests for individual items. Independent 2-tailed *t* tests were performed to test for differences in mean scores per the UTAUT construct. In total, 16 respondents participated in the interviews (10 users and 6 nonusers of apps for T2DM). We performed content analysis using a deductive approach on all transcripts, guided by the UTAUT.

**Results:**

Regarding actual use, 55.3% (57/103) were nonusers and 44.7% (46/103) were users of apps for T2DM. The main driver for the use of apps was the belief that using apps for managing diabetes would result in better personal health and well-being. The time and energy required to keep track of the data and understand the app were mentioned as barriers. Mean scores were significantly higher among users compared with nonusers of apps for T2DM for the constructs performance expectancy (4.06, SD 0.64 vs 3.29, SD 0.89; *P*<.001), effort expectancy (4.04, SD 0.62 vs 3.50, SD 0.82; *P*<.001), social influence (3.59, SD 0.55 vs 3.29, SD 0.54; *P*=.007), and facilitating conditions (4.22, SD 0.48 vs 3.65, SD 0.70; *P*<.001). On the basis of 16 in-depth interviews, it was recognized that health care professionals play an important role in supporting patients with T2DM in using apps. However, respondents noticed that their health care professionals were often not supportive of the use of apps for managing diabetes, did not show interest, or did not talk about apps. Reimbursement by insurance companies was mentioned as a missing facilitator.

**Conclusions:**

Empowering health care professionals’ engagement is of utmost importance in supporting patients with T2DM in the use of apps. Insurance companies can play a role in facilitating the use of diabetes apps by ensuring reimbursement.

## Introduction

### Background

Type 2 diabetes mellitus (T2DM) is a serious public health concern globally. The prevalence of T2DM is increasing at a rapid pace in developed regions, such as Western Europe including the Netherlands, and causes a substantial economic burden [1-3]. Various lifestyle factors are important for the development of T2DM [4,5]. To manage diabetes sufficiently, adherence to regular physical activity and a healthy diet are important. Several studies have reported the positive effects of lifestyle interventions, including regular physical activity and healthy food intake, on the stabilization of blood glucose levels and health status of patients with T2DM [5-10].

### Mobile Health Apps

T2DM requires self-management and support. Self-monitoring of blood glucose levels, food intake, and physical activity can support the self-management of patients with T2DM. In recent years, there has been an increase in the development and availability of technologies for diabetes self-monitoring, especially mobile health apps [11], for example, an app to integrate and keep track of blood glucose levels in combination with data regarding physical activity and food intake. These apps have considerable potential to support diabetes self-management and have a positive effect on a person’s lifestyle [12-14]. However, studies have shown that the uptake of apps for managing diabetes is rather low [15-18]. Insight is needed into how apps can be integrated into diabetes self-management care [19]. Research regarding the acceptance of apps for managing diabetes among patients is important for their successful implementation. Several quantitative and qualitative studies have been performed to gain further insight into the acceptance of apps for managing diabetes among patients with diabetes [20-24]. Zhang et al [22] investigated predictors of the intention to use apps for managing diabetes using a web-based questionnaire. They found that performance expectancy (ie, perceived usefulness) and social influence were the most important determinants of the intention to use apps for managing diabetes. Torbjørnsen et al [23] conducted interviews to obtain an in-depth understanding of users’ acceptance of a mobile app for diabetes. They found that users’ acceptance of mobile apps for diabetes self-management differed. Regular use of an app could be useful (supportive and educational) but could also become a burden, requiring too much time and not contributing enough to the effort needed to change lifestyles. Furthermore, Torbjørnsen et al [23] concluded that both practical (ie, usability and utility) and social aspects (ie, attitude and shared understanding) are important for the acceptability of mobile apps for diabetes. Jeffrey et al [24] conducted semistructured phone interviews among patients with T2DM and found that a lack of knowledge and awareness of apps as health care tools was one of the barriers.

### Unified Theory of Acceptance and Use of Technology

The unique aspect of this study is the use of an explanatory sequential design (mixed methods) among both users and nonusers. To explore the actual use of apps for T2DM and gain greater insight into the main barriers and drivers, a web-based questionnaire using the Unified Theory of Acceptance and Use of Technology (UTAUT) was deployed along with in-depth interviews. The UTAUT is a unified model that was developed by Venkatesh et al [25] and is commonly used globally in studies regarding health technology acceptance. The UTAUT is based on the Social Cognitive Theory with a combination of 8 prominent information technology acceptance research models (Theory of Reasoned Action, Theory of Planned Behavior, Technology Acceptance Model, Motivation Model, a model combining the Technology Acceptance Model and the Theory of Planned Behavior, Model of Personal Computer Use, Diffusion of Innovation theory, and Social Cognitive Theory). Validation by Venkatesh et al [25] showed that the UTAUT accounts for 70% of the variance in behavioral intention to use and about 50% in actual use. On the basis of the results of this study, recommendations are described for future research and the integration of apps with diabetes self-management care.

### Aim

The aim of this study is to investigate the actual use and the barriers and drivers among users and nonusers of mobile health apps for T2DM. The use of a mixed methods approach is different compared with most previous studies using the UTAUT and investigating the use of eHealth among patients with diabetes. Most studies have applied quantitative research methods, and some have conducted interviews. The addition of in-depth interviews based on the quantitative findings is therefore different from previous studies and could show more explicitly where patients with T2DM need support and how the barriers and drivers influence their use of apps to self-manage diabetes. Furthermore, the focus on patients with T2DM is rather novel, as is the inclusion of nonusers of apps. These nonusers were included to understand their view toward apps, and the hypothesis of this study is that some of them are not unwilling to use apps but rather are unaware of the availability.

## Methods

### Research Design

An explanatory sequential design [26], using a mixed methods approach, was applied to assess the actual use, barriers, and drivers among users and nonusers of apps for T2DM. This study started with the collection and analysis of a web-based questionnaire using Qualtrics software (Qualtrics International Inc). The web-based questionnaire was designed and reported based on the Checklist for Reporting Results of Internet E-Surveys (CHERRIES) [27]. The web-based questionnaire was pretested for usability and technical functionality before fielding. The qualitative data were used to design the interview guide (Multimedia Appendix 1) so that it follows from the quantitative phase and provides the opportunity to obtain in-depth information. The following in-depth semistructured phone interviews lasted between 25 and 50 minutes, audiotapes were made, and field notes were taken. Ethical approval was obtained from the University of Twente Ethical Committee (reference number 201213). Participants were informed about the purpose of the study and length and time of the questionnaire and which data were stored and where before the start of the web-based questionnaire. They provided web-based consent and were informed about their right to withdraw at any time. Data were anonymized, confidentiality was maintained, and the data will be retained for 10 years.

### Recruitment Strategy and Sample Size

The study population included patients with T2DM aged ≥16 years spread across the Netherlands. Recruitment for the web-based questionnaire was performed using convenience sampling by publishing the link on web-based platforms for patients with T2DM, in the newsletter of the Dutch Diabetes Association, and social media. A total of 183 respondents completed the questionnaire. In total, 80 respondents did not complete the questionnaire sufficiently (missing data on actual use or barriers and drivers) and were excluded. Questionnaire data were analyzed from 103 respondents. At the end of the questionnaire, participants were asked to leave their phone number if they were willing to participate in a follow-up phone interview. A total of 16 respondents participated in the phone interviews; 10 were users and 6 were nonusers of apps to manage T2DM. Data were collected between July and December 2020.

### Measures

#### Actual Use

In the web-based questionnaire, 1 item was included to measure the actual use of apps for T2DM, namely, “Do you use apps for T2DM specifically (for example health-apps to get insight into your blood glucose levels or a digital coach for support in daily life with diabetes)?”

#### Barriers and Drivers

The well-established UTAUT has been demonstrated to be a reliable theoretical framework for studying barriers and drivers for users’ acceptance of information technology [25,28]. The UTAUT has been used in many studies globally [29]. The UTAUT includes 4 key constructs (ie, performance expectancy, effort expectancy, social influence, and facilitating conditions). Focusing on diabetes apps, performance expectancy is the level to which an individual believes that an app will help them gain health performance, whereas effort expectancy is the level of ease associated with the use of an app. Social influence is the degree to which an individual finds it important that others believe they should use the app, whereas facilitating conditions are the measure of infrastructural support available for app use [25]. The questionnaire used in this study was drawn based on the classification of the Flemish UTAUT questionnaire [30,31]. The scales consisted of the 4 key constructs: (1) performance expectancy (4 items), (2) effort expectancy (3 items), (3) social influence (4 items), and (4) facilitating conditions (3 items). Furthermore, the constructs anxiety (2 items), trust in data security (2 items), and knowledge (2 items) were added [30]. For each item, respondents answered on a 5-point Likert scale ranging from strongly disagree to strongly agree. The interview guide (Multimedia Appendix 1) was based on the findings of the quantitative data collected.

### Data Analysis

The questionnaire data were analyzed using IBM SPSS Statistics (version 25.0). Descriptive statistics were performed for the actual use of apps among patients with T2DM and the individual items of the UTAUT. Differences between users and nonusers were tested using chi-square tests for the individual items. Independent 2-tailed *t* tests were performed to test for differences in mean scores per UTAUT construct. Therefore, 3 items were reverse-coded: *Using apps would cost me a lot of time and energy (effort expectancy)*, *Other people would think bad of me if I used apps (social influence)*, and *When I think about using apps, I fear that confidential information could end up in the wrong hands (trust in data security)*. To analyze the qualitative data, interviews were transcribed verbatim. Content analysis with a deductive approach was performed on all transcripts guided by the UTAUT [32]. The UTAUT was used in this analysis to align the integration of quantitative and qualitative data. Data management was performed using the NVivo (version 11) software package. During the organizing phase of the analysis, a matrix was developed comprising the components of performed expectancy, effort expectancy, social influence, facilitating conditions, anxiety, and trust in data security and knowledge. Categories were created within each component of the analysis matrix (Multimedia Appendix 2).

### Validity

Credibility was established using several procedures [33]. Method triangulation was performed using multiple data collection methods, namely questionnaires and individual interviews with audio recording. Researcher triangulation was achieved by 3 researchers (MB, CMvL, and TJJO) who performed the interviews and read and compared the findings. Peer debriefing took place during weekly meetings with the project team, where scientific and organizational aspects were discussed. When data collection was complete, a member check was performed by sharing an infographic with the respondents. A thick description was developed for transferability [33], which included the sampling method, recruitment, data collection, questionnaire and interviewing method, and analysis process.

## Results

### Participants

Of the respondents who completed the questionnaire, 55.3% (57/103) were men and 41.7% (43/103) were women (Table 1). The mean age was 69 years (SD 10.8; range 27-90 years). Most respondents (97/103, 94.2%) were of Dutch origin, and 28.2% (29/103) had a lower education, 17.5% (18/103) had an intermediate education, and 50.5% (52/103) had a higher education. Most respondents (84/103, 81.6%) had been diagnosed with T2DM ≥10 years ago. Demographic characteristics of the respondents from the interviews (n=16) were comparable with the demographic characteristics of the overall respondents from the questionnaire (N=103; Table 1).

**Table 1:** Demographic characteristics of respondents.

Characteristics			Questionnaire (N=103), n (%)		Interviews (n=16), n (%)
**Gender**					
	Male	57 (55.3)		10 (62.5)	
	Female	43 (41.7)		6 (37.5)	
	Unknown	3 (2.9)		0 (0)	
**Age (years)**					
	<30	1 (1)		0 (0)	
	30-49	5 (4.9)		2 (12.5)	
	50-69	42 (40.8)		7 (43.8)	
	≥70	51 (49.5)		7 (43.8)	
	Unknown	4 (3.9)		0 (0)	
**Ethnicity**					
	Dutch	97 (94.2)		16 (100)	
	Non-Dutch	3 (2.9)		0 (0)	
	Unknown	3 (2.9)		0 (0)	
**Educational level^a^**					
	Low	29 (28.2)		5 (31.3)	
	Intermediate	18 (17.5)		2 (12.5)	
	High	52 (50.5)		9 (56.3)	
	Unknown	4 (3.9)		0 (0)	
**Marital status**					
	Single	20 (19.4)		5 (31.3)	
	Married or cohabiting	77 (74.8)		10 (62.5)	
	Other	3 (2.9)		1 (6.3)	
	Unknown	3 (2.9)		0 (0)	
**Disease duration (years)**					
	<1	3 (2.9)		0 (0)	
	1-3	2 (1.9)		0 (0)	
	4-6	2 (1.9)		0 (0)	
	7-9	9 (8.7)		3 (18.8)	
	≥10	84 (81.6)		13 (81.3)	
	Unknown	3 (2.9)		0 (0)	

^a^Lower education (ie, primary education, lower general or lower vocational education, or less), intermediate (ie, secondary general or vocational education), and higher education (ie, higher professional education or university).

### Actual Use

On the basis of the questionnaire, 55.3% (57/103) were nonusers and 44.7% (46/103) were users of apps for managing T2DM. Of the 46 respondents who reported using apps for T2DM, 35 (78%) had used them for ≥12 months.

### Barriers and Drivers

#### Performance Expectancy

Most of the app users, that is, 74% (34/46) to 85% (39/46), expected benefits for personal health and well-being because of the use of apps for managing T2DM (Multimedia Appendix 3). In total, 85% (39/46) of users believed that apps would help them deal with their health problems and 78% (36/46) believed that apps would help them reduce their health problems. A participant stated, by using an app to assist with diabetes care, *“*I hoped that the app would help me achieve better blood sugar levels, because it has the ability to measure more often and react immediately when necessary.*”* Among nonusers, there were more doubts whether using apps would result in improving personal well-being or reducing health problems, that is, 46% (26/57) believed that using apps would improve their personal well-being or would help them deal with their health problems. However, in the interviews, nonusers also believed that an app that shows or registers blood glucose levels would be beneficial. Most nonusers saw benefits in the use of apps for an active and healthy lifestyle: “It should give me a reminder that I have to go out and move a little.”

#### Effort Expectancy

Regarding the expected ease of app use, most (42/46, 91%) users (strongly) agreed that the use of apps would be an easy task, and half of the nonusers (30/57, 53%) thought that apps would be clear and easily comprehensible to them (Multimedia Appendix 3). Time and energy were the main topics in the in-depth interviews. It would cost time to keep track of the data, but it would also cost time and energy to understand the app: “A more user-friendly app would be desirable.” Owing to the complexity of some apps, “many are not interested and I do not want to spend so much energy learning how to use it.” This was agreed upon by one of the users, but he also stated that “initially it took more effort to measure and register all food, after that it became easy to keep track of my food intake.” Furthermore, all participants agreed that app use should be a joint task involving their health care professional. Although the use of apps may seem time- or energy-consuming, most users experienced less effort in real life to manage their diabetes sufficiently: “Based on the blood sugar level, my weight, and carbohydrate intake, the app communicates how much insulin I need.”

#### Social Influence

Regarding the influence of the social environment, a minority of respondents thought that their general practitioner would recommend the use of apps, that is, 37% (17/46) of users and 19% (11/57) of nonusers (Multimedia Appendix 3). Findings from the in-depth interviews showed that respondents wanted to use apps and acquire data together with their health care professional. They agreed that, “as a layman I can see if my blood sugar level is too high, but my knowledgeable physician has to take over at that point.” However, most respondents said that health care professionals were not supportive and they did not talk about apps or acquired data or that respondents did not ask them about apps. Some respondents acknowledged that health care professionals could not know all apps: “I understand that it is impossible to give advice about these apps, they do not know all apps on the market, which is best for me and how all apps function.” In addition to health care professionals, family and friends are also important. In all interviews, the respondents talked about support from family and friends. On the one hand, regarding self-managing their diabetes sufficiently and, on the other hand, about the use of apps: “My grandchildren love the funny little man in the app, because if my glucose is too high, he says ‘fie, fie, fie’ or if it is too low, he says ‘eat, eat, eat’.” Whereas health care professionals and family and friends are very important to all respondents, regular contact with other diabetics is seen as not so important in daily life. However, there was 1 respondent who started using a diabetes app owing to a recommendation by another member of the diabetes association.

#### Facilitating Conditions

Most respondents (98/103, 95.1%) had a computer or smartphone with internet access and could use apps (Multimedia Appendix 3). Among the nonusers, almost half of the respondents (26/57, 46%) were convinced that they possessed the knowledge to use apps, and 37% (21/57) had someone available to support them in case of problems. The interviews showed that patients expect health care professionals to facilitate their use of apps. Although almost all respondents had a computer or smartphone and none had experienced failure in using technology, another missing facilitator was financing or reimbursement by insurance companies: “People with Type 2 Diabetes do not get reimbursement for all these apps, now I have to pay for it myself and that is of course too much.”

#### Anxiety

In total, 82 (79.6%) of the 103 respondents were not afraid of making irrevocable mistakes when using the internet or apps (Multimedia Appendix 3). In addition, most users (37/46, 80%) and nonusers (36/57, 63%) strongly disagreed with the statement that “the internet feels like something threatening.” However, interviews indicated that some of the nonusers were afraid of the speed of innovations coming into the market. There was a wide range of responses regarding how the respondents reacted to using apps. Some respondents were anxious about touching the wrong key in an app, whereas others did not mind if something went wrong. Other respondents thought it was logical, and mistakes were impossible: “The app does not bite, if I do something wrong what can happen?” Furthermore, they felt threatened by considering the influence of the app on their life: “I do not want my life to be run by an app.”

#### Trust in Data Security

Most users (35/46, 76%) and nonusers (37/57, 65%) trust that the information they provide when using apps is handled with strictest confidence (Multimedia Appendix 3). However, 19% (11/57) of nonusers feared that confidential information could end up in the wrong hands. Similar findings were found in the in-depth interviews. All respondents handed over the data acquired while using an app to their health care professional. All respondents who used apps to keep track of their diabetes and made graphs of their blood glucose levels shared this during their regular appointments: “That is super, because she can immediately see a visualization of my values and understand that I sometimes like to eat a bar of chocolate and how my body reacts.” However, there were health care professionals who were not interested in the data. Most respondents were not concerned with privacy issues, but one nonuser distrusted all apps, because “there are too many privacy risks in using smartphones, and too much pressure from software companies.”

#### Knowledge

Approximately three-quarters of the users (34/46, 74%) and 42% (24/57) of the nonusers knew what to expect from apps (Multimedia Appendix 3). Although the interviews showed that none of the nonusers knew any useful apps to support the management of T2DM, most respondents were open to trying apps to support them in daily life, “I am willing to try new innovations, that much I know!” In addition, not all have the knowledge needed or interest in using apps: “It is too difficult for me to learn more about these new technologies, I do not know how to use them, and it costs me a lot of effort to try to understand them.”

### Differences in Perceptions Between Users and Nonusers

Table 2 shows the differences in mean scores between users and nonusers of apps for T2DM per the UTAUT construct. Mean scores were significantly higher among users of apps for managing T2DM for the constructs performance expectancy, effort expectancy, facilitating conditions, and knowledge compared with nonusers (*P*<.001). In addition, users scored significantly higher regarding social influence (*P*=.007) compared with nonusers, whereas mean scores regarding anxiety were significantly lower among users compared with nonusers (*P*=.003). Finally, the mean scores for trust in data security were significantly higher among users compared with nonusers (*P*=.02).

**Table 2:** Differences between users and nonusers of apps for type 2 diabetes mellitus per the Unified Theory of Acceptance and Use of Technology construct.

Construct	Nonusers (n=57), mean (SD)	Users (n=46), mean (SD)	Values	
			*t* test (*df*)	*P* value
Performance expectancy	3.29 (0.89)	4.06 (0.64)	5.15 (101)	<.001
Effort expectancy	3.50 (0.82)	4.04 (0.62)	3.71 (101)	<.001
Social influence	3.29 (0.54)	3.59 (0.55)	2.77 (101)	.007
Facilitating conditions	3.65 (0.70)	4.22 (0.48)	4.94 (101)	<.001
Anxiety	2.11 (0.86)	1.65 (0.71)	−2.93 (101)	.003
Trust in data security	3.48 (0.75)	3.85 (0.74)	2.48 (101)	.02
Knowledge	3.28 (0.97)	4.11 (0.74)	4.91 (101)	<.001

## Discussion

### Summary of Findings

The aim of this study was to investigate the actual use and the barriers and drivers among users and nonusers of mobile health apps for T2DM. This study showed that the main drivers were the belief that using apps for managing diabetes will result in better personal health and well-being and that, by sharing their data with health care professionals, users will receive improved support. The barriers included time and energy to keep track of data and understand the app, lack of support by health care professionals, and no financial support or reimbursement by insurance companies. Nonusers had more doubts regarding the improved support provided by apps and showed more anxiety and distrust toward the use of apps. However, most nonusers were willing to try apps to help manage their diabetes.

### Reflection With the Literature

#### Performance Expectancy

This study showed that performance expectancy (ie, the belief that using apps for managing diabetes will help to deal with health problems and improve personal well-being) was one of the main drivers for the use of apps for managing diabetes. Performance expectancy, next to social influence, was also found in other studies as one of the drivers for the intention to use apps for managing diabetes [22-24]. In line with the interviews, Torbjørnsen et al [23] described that routine use of apps for managing diabetes could provide a meaningful overview of blood glucose levels, diet, and activity and provide fresh insight into self-management. In a study by Jeffrey et al [24], most of the participants concluded that app use improved their diabetes management. Additional measurements help patients with T2DM gain insight into their disease and allow them to react immediately when necessary.

#### Effort Expectancy

In line with previous studies regarding effort expectancy (ie, the level of ease associated with the use of apps for managing diabetes), respondents stated that it takes time to keep track of data and understand an app. Regular measurements of parameters such as physical activity and food intake, in addition to blood glucose levels, are time-consuming in a busy everyday life and can be stressful [23]. Hence, the use of apps for managing diabetes has an impact on the daily life of patients with T2DM. If the impact is noticed by users and positively changes behavior, persistence in use will increase [21]. However, the use of these apps should be easy [20,21], and automation is desirable to reduce the time required to perform tasks [21]. Both Scheibe et al [20] and the nonusers in our study did not expect any benefit from apps and expected the effort required to be so high that it would not be worth starting to use apps.

#### Social Influence

Social influence is an important factor that contributes to the intention to use apps [34]. On the one hand, patients stated that health care professionals are an important source of support when it comes to using apps and want to share their app use and data [21,35,36]. Stühmann et al [35] conducted a cross-sectional survey in Germany and found that participants who obtained health advice from a physician were more likely to use health apps compared with those who received no advice on any health behavior. On the other hand, patients are dissatisfied with the supervision or involvement of their professional in the use of apps for diabetes self-care. In our study, respondents noticed that their health care professionals were often not supportive of the use of apps for managing diabetes, did not show interest, or did not talk about apps or acquired data. A lack of knowledge about apps may be the main barrier for health care professionals. Hence, transfer of knowledge (ie, information and education) regarding apps for managing diabetes should not only focus on patients with T2DM but also focus on health care professionals. They can intervene as social agents to explain the use, usefulness, and benefits of apps [37]. Research has shown that support from health care professionals empowers patients to use apps and improves diabetes self-management [36]. Therefore, apps can have a positive effect on the relationship between health care professionals and patients [38]. However, a higher workload for health care professionals could also negatively affect the relationship [36]. Furthermore, information about the use and experience of apps for managing diabetes of peers with T2DM can be a motivator in the acceptance of app use. In this study, regular contact with other patients with T2DM seemed of little importance, but patients with T2DM could be motivated by peers and learn from others’ experiences regarding app use.

#### Facilitating Conditions

Regarding facilitating conditions, both our study and previous studies have shown that nonusers have limited knowledge about what to expect from apps, where to find them, or how to use them [24]. Using apps for managing diabetes requires not only knowledge but also digital competences of end users in order to become familiar with the use of apps and to integrate them into daily life. The need for competence and digital literacy has been acknowledged in many other studies [20,21,24,39,40]. Thorsen et al [40] concluded that the implementation of health technology among patients with T2DM should be based on a comprehensive consideration of readiness for health technology. Reimbursement by insurance companies was mentioned as a missing facilitator, as often the financial resources are lacking among patients with T2DM in the Netherlands. Insurance companies can play a role in facilitating the use of apps for managing diabetes, for example, by assuring reimbursement despite the availability of financial resources. Besides reimbursement, incentives are a common mechanism applied in mobile health apps for managing diabetes for engaging, empowering, and retaining patients [41]. Finally, trust in data security is a major issue, especially among nonusers. They expected more privacy risks and had a higher anxiety level when considering app use. Similar to the findings of Cimperman et al [37], focus should be placed on portraying the apps as secure and easy to use. This could be a key factor in the acceptance of all patients with T2DM. The limitation of problems with connectivity [24] was not a barrier experienced by respondents in our study.

### Strengths and Limitations

One strength of this study is the triangulation method of applying an explanatory sequential design. Hence, rich data were collected, which provided in-depth insight into the actual use of mobile health apps among patients with T2DM and the main barriers and drivers for use. Second, the well-established UTAUT model was used as the base for the questionnaire [25]. The interviews provided in-depth insight and a more explicit description of each UTAUT item. Finally, the study was performed, and data were analyzed by 3 researchers, which contributed to researcher triangulation. There were some limitations to this study. A possible bias considers digital literacy and respondents’ interest in technology. Most respondents were of Dutch origin and had a higher educational level. Barriers to and motivators for the use of mobile health apps for managing diabetes may differ among specific subgroups (ie, people of non-Dutch ethnicity or people with a lower level of education). Approximately half of the respondents were app users for T2DM, which is a rather high percentage compared with other studies [15-18]. It might be the case that most respondents who filled out the questionnaire had a personal interest in this topic. Furthermore, the response and completion rates (103/180, 57.2%) of respondents who completed the web-based questionnaire were relatively small.

### Recommendations

The literature, as well as this study, has shown that apps may increase diabetes self-management. It is important to integrate apps for managing diabetes into the daily practice of diabetes self-management care, with health care professionals playing an important role. On the basis of the findings of this study, we recommend that health care professionals get more involved and acquire relevant knowledge about mobile health apps specifically for patients with diabetes. The technology involved assists patients with T2DM to self-manage diabetes and assists professionals in supporting clients in their self-management. Currently, multiple apps are available for the management of diabetes. Therefore, it is difficult to know which apps would be most beneficial to whom and why. A study to investigate patient experiences among specific subgroups (ie, people of non-Dutch ethnicity or people with a lower level of education) in the use of different apps for managing diabetes is the next step for research. In addition, training patients with T2DM and professionals regarding the availability and use of apps is recommended. Important topics for such training include digital knowledge and competencies, learning about apps, how to track data, and how to read and use the collected data. The implementation of apps for managing diabetes in daily practice is complex. This study provides recommendations that focus on the main drivers and barriers. However, other factors (such as the type of organization, availability, and type of patients) also play a role in the implementation process.

### Conclusions

One of the main drivers for use was the belief that using apps for managing diabetes would result in better personal health and well-being. The time and energy required to keep track of the data and understand the app were mentioned as barriers. Patients with T2DM stated that health care professionals’ engagement is of the utmost importance in supporting them in app use. In addition, patients stated that insurance companies can play a role in facilitating the use of diabetes apps, for example, by assuring reimbursement. Further research should focus on the evaluation of patients’ experiences with different apps for managing diabetes, how to integrate apps into diabetes self-management care, and investigating barriers and motivators in the use of mobile health apps for the management of diabetes among specific subgroups (ie, people of non-Dutch ethnicity or people with a lower level of education).

## Appendix

Multimedia Appendix 1 Interview guide (English translation of Dutch original).

Multimedia Appendix 2 Coding matrix.

Multimedia Appendix 3 Quantitative results per the Unified Theory of Acceptance and Use of Technology item.

## Abbreviations

T2DM: type 2 diabetes mellitus
UTAUT: Unified Theory of Acceptance and Use of Technology
