# Supplementary material for: Barriers and Drivers Regarding the Use of Mobile Health Apps Among Patients With Type 2 Diabetes Mellitus in the Netherlands: Explanatory Sequential Design Study
Source: JMIR Diabetes. 2022 Jan 27;7(1):e31451. doi: 10.2196/31451 (PMC8832276; doi:10.2196/31451)
Supplement: Multimedia Appendix 1 [file diabetes_v7i1e31451_app1.docx]

# **Multimedia Appendix 1.** Interview guide (English translation of Dutch original)

1. Can you introduce yourself? Can you elaborate on your life with type 2 diabetes?
2. Do you use apps in your life with diabetes?
   1. *If yes:* Which apps do you use?
       *If no*: Do you know any apps that could support you with diabetes?
   2. *If yes:* How do you use these apps?
       *If no:* Are you interested in using apps?
   3. Do you need help in using apps?
3. Which professionals assist you in the management of diabetes type 2?
   1. Have you spoken with them about apps?
   2. Will you share data obtained with apps with your healthcare professionals?
4. Which expectations do you have considering the future and your life with diabetes?
   1. What would you wish for the future?
