# Supplementary figures and images for: Barriers and Drivers Regarding the Use of Mobile Health Apps Among Patients With Type 2 Diabetes Mellitus in the Netherlands: Explanatory Sequential Design Study
Source: JMIR Diabetes. 2022 Jan 27;7(1):e31451. doi: 10.2196/31451 (PMC8832276; doi:10.2196/31451)

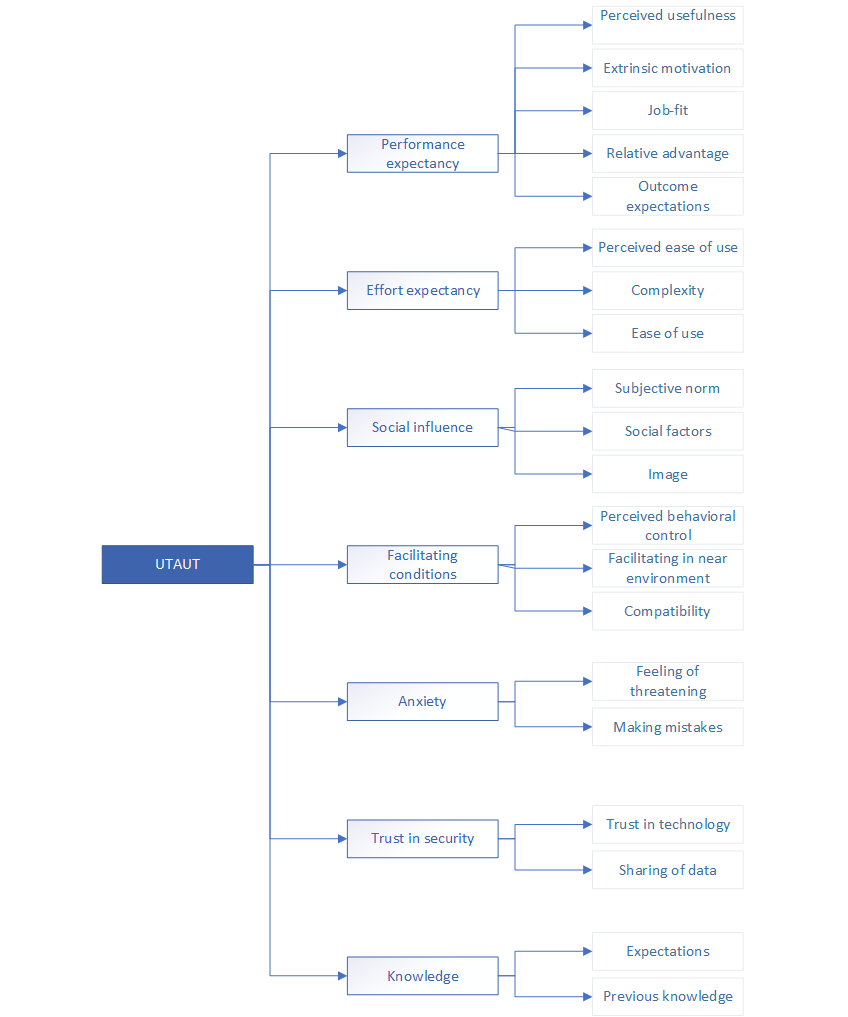

Supplement: Multimedia Appendix 2 [file diabetes_v7i1e31451_app2.png]
