# Supplementary material for: Barriers and Drivers Regarding the Use of Mobile Health Apps Among Patients With Type 2 Diabetes Mellitus in the Netherlands: Explanatory Sequential Design Study
Source: JMIR Diabetes. 2022 Jan 27;7(1):e31451. doi: 10.2196/31451 (PMC8832276; doi:10.2196/31451)
Supplement: Multimedia Appendix 3 [file diabetes_v7i1e31451_app3.docx]

**Multimedia Appendix 3.** Quantitative results per the Unified Theory of Acceptance and Use of Technology item.

|  | | | Overall (N=103), (n, %) | Nonusers (n=57), (n, %) | Users (n=46), (n, %) |
| --- | --- | --- | --- | --- | --- |
| **Performance expectancy** | | | | | |
|  | **“Using apps would reduce my health problems.” (*P*=.002)** | | | | |
|  |  | (Strongly) disagree (score 1-2) | 13 (12.6) | 11 (19.3) | 2 (4.3) |
|  |  | Neutral (score 3) | 31 (30.1) | 22 (38.6) | 9 (19.6) |
|  |  | (Strongly) agree (score 4-5) | 59 (57.3) | 24 (42.1) | 35 (76.1) |
|  | **“Using apps would improve my personal well-being.” (*P*=.002)** | | | | |
|  |  | (Strongly) disagree (score 1-2) | 10 (9.7) | 10 (17.5) | 0 (0) |
|  |  | Neutral (score 3) | 33 (32.0) | 21 (36.8) | 12 (26.1) |
|  |  | (Strongly) agree (score 4-5) | 60 (58.3) | 26 (45.6) | 34 (73.9) |
|  | **“Apps can help me reduce my health problems.” (*P*<.001)** | | | | |
|  |  | (Strongly) disagree (score 1-2) | 12 (11.7) | 10 (17.5) | 2 (4.3) |
|  |  | Neutral (score 3) | 34 (33.0) | 26 (45.6) | 8 (17.4) |
|  |  | (Strongly) agree (score 4-5) | 57 (55.3) | 21 (36.8) | 36 (78.3) |
|  | **“Using apps would help me deal with my health problems.”** **(*P*<.001)** | | | | |
|  |  | (Strongly) disagree (score 1-2) | 11 (10.7) | 11 (19.3) | 0 (0) |
|  |  | Neutral (score 3) | 27 (26.2) | 20 (35.1) | 7 (15.2) |
|  |  | (Strongly) agree (score 4-5) | 65 (63.1) | 26 (45.6) | 39 (84.8) |
| **Effort expectancy** | | | | | |
|  | **“Using apps would cost me a lot of time and energy.”** **(*P*=.24)** | | | | |
|  |  | (Strongly) disagree (score 1-2) | 56 (54.4) | 27 (47.4) | 29 (63.0) |
|  |  | Neutral (score 3) | 25 (24.3) | 17 (29.8) | 8 (17.4) |
|  |  | (Strongly) agree (score 4-5) | 22 (21.4) | 13 (22.8) | 9 (19.6) |
|  | **“Using apps would be an easy task for me.” (*P*=.001)** | | | | |
|  |  | (Strongly) disagree (score 1-2) | 9 (8.7) | 8 (14.0) | 1 (2.2) |
|  |  | Neutral (score 3) | 18 (17.5) | 15 (26.3) | 3 (6.5) |
|  |  | (Strongly) agree (score 4-5) | 76 (73.8) | 34 (59.6) | 42 (91.3) |
|  | **“Apps would be clear and easily comprehensible to me.” (*P*=.001)** | | | | |
|  |  | (Strongly) disagree (score 1-2) | 9 (8.7) | 8 (14.0) | 1 (2.2) |
|  |  | Neutral (score 3) | 24 (23.3) | 19 (33.3) | 5 (10.9) |
|  |  | (Strongly) agree (score 4-5) | 70 (68.0) | 30 (52.6) | 40 (87.0) |
| **Social influence** | | | | | |
|  | **“People around me would recommend me to use apps.” (*P*=.70)** | | | | |
|  |  | (Strongly) disagree (score 1-2) | 31 (30.1) | 19 (33.3) | 12 (26.1) |
|  |  | Neutral (score 3) | 41 (39.8) | 21 (36.8) | 20 (43.5) |
|  |  | (Strongly) agree (score 4-5) | 31 (30.1) | 17 (29.8) | 14 (30.4) |
|  | **“My general practitioner would recommend that I use apps.” (*P*=.12)** | | | | |
|  |  | (Strongly) disagree (score 1-2) | 18 (17.5) | 12 (21.1) | 6 (13.0) |
|  |  | Neutral (score 3) | 57 (55.3) | 34 (59.6) | 23 (50.0) |
|  |  | (Strongly) agree (score 4-5) | 28 (27.2) | 11 (19.3) | 17 (37.0) |
|  | **“People in my area would use apps if they had Type 2 Diabetes.” (*P*=.045)** | | | | |
|  |  | (Strongly) disagree (score 1-2) | 16 (15.5) | 12 (21.1) | 4 (8.7) |
|  |  | Neutral (score 3) | 50 (48.5) | 30 (52.6) | 20 (43.5) |
|  |  | (Strongly) agree (score 4-5) | 37 (35.9) | 15 (26.3) | 22 (47.8) |
|  | **“Other people would think bad of me if I used apps.” (*P*=.35)** | | | | |
|  |  | (Strongly) disagree (score 1-2) | 89 (86.4) | 47 (82.5) | 42 (91.3) |
|  |  | Neutral (score 3) | 12 (11.7) | 9 (15.8) | 3 (6.5) |
|  |  | (Strongly) agree (score 4-5) | 2 (1.9) | 1 (1.8) | 1 (2.2) |
| **Facilitating conditions** | | | | | |
|  | **“I have a computer or smartphone with internet access and could use apps.” (*P*=.12)** | | | | |
|  |  | (Strongly) disagree (score 1-2) | 1 (1.0) | 1 (1.8) | 0 (0.0) |
|  |  | Neutral (score 3) | 4 (3.9) | 4 (7.0) | 0 (0.0) |
|  |  | (Strongly) agree (score 4-5) | 98 (95.1) | 52 (91.2) | 46 (100.0) |
|  | **“In case of technical problems with apps, someone could provide me with technical support.” (*P*=.80)** | | | | |
|  |  | (Strongly) disagree (score 1-2) | 21 (20.4) | 16 (28.1) | 5 (10.9) |
|  |  | Neutral (score 3) | 37 (35.9) | 20 (35.1) | 17 (37.0) |
|  |  | (Strongly) agree (score 4-5) | 45 (43.7) | 21 (36.8) | 24 (52.2) |
|  | **“I have the necessary technical knowledge to use apps.” (*P*<.001)** | | | | |
|  |  | (Strongly) disagree (score 1-2) | 11 (10.7) | 11 (19.3) | 0 (0.0) |
|  |  | Neutral (score 3) | 25 (24.3) | 20 (35.1) | 5 (10.9) |
|  |  | (Strongly) agree (score 4-5) | 67 (65.0) | 26 (45.6) | 41 (89.1) |
| **Anxiety** | | | | | |
|  | **“The Internet sometimes feels like something threatening.” (*P*=.06)** | | | | |
|  |  | (Strongly) disagree (score 1-2) | 73 (70.9) | 36 (63.2) | 37 (80.4) |
|  |  | Neutral (score 3) | 21 (20.4) | 13 (22.8) | 8 (17.4) |
|  |  | (Strongly) agree (score 4-5) | 9 (8.7) | 8 (14.0) | 1 (2.2) |
|  | **“I am afraid of making an irrevocable mistake when using the Internet.” (*P*=.08)** | | | | |
|  |  | (Strongly) disagree (score 1-2) | 82 (79.6) | 43 (75.4) | 39 (84.8) |
|  |  | Neutral (score 3) | 15 (14.6) | 8 (14.0) | 7 (15.2) |
|  |  | (Strongly) agree (score 4-5) | 6 (5.8) | 6 (10.5) | 0 (0.0) |
| **Trust in data security** | | | | | |
|  | **“When using apps, I trust that any information I provide is handled in strictest confidence.” (*P*=.43)** | | | | |
|  |  | (Strongly) disagree | 10 (9.7) | 7 (12.3) | 3 (6.5) |
|  |  | Neutral (score 3) | 21 (20.4) | 13 (22.8) | 8 (17.4) |
|  |  | (Strongly) agree (score 4-5) | 72 (69.9) | 37 (64.9) | 35 (76.1) |
|  | **“When I think about using apps, I fear that confidential information could end up in the wrong hands.” (*P*=.04)** | | | | |
|  |  | (Strongly) disagree (score 1-2) | 49 (47.6) | 21 (36.8) | 28 (60.9) |
|  |  | Neutral (score 3) | 39 (37.9) | 25 (43.9) | 14 (30.4) |
|  |  | (Strongly) agree (score 4-5) | 15 (14.6) | 11 (19.3) | 4 (8.7) |
| **Knowledge** | | | | | |
|  | **“I have a clear idea of what to expect from apps.” (*P*=.004)** | | | | |
|  |  | (Strongly) disagree (score 1-2) | 11 (10.7) | 9 (15.8) | 2 (4.3) |
|  |  | Neutral (score 3) | 34 (33.0) | 24 (42.1) | 10 (21.7) |
|  |  | (Strongly) agree (score 4-5) | 58 (56.3) | 24 (42.1) | 34 (73.9) |
|  | **“I already have some knowledge of apps.” (*P*<.001)** | | | | |
|  |  | (Strongly) disagree (score 1-2) | 15 (14.6) | 14 (24.6) | 1 (2.2) |
|  |  | Neutral (score 3) | 21 (20.4) | 17 (29.8) | 4 (8.7) |
|  |  | (Strongly) agree (score 4-5) | 67 (65.0) | 26 (45.6) | 41 (89.1) |
